# Rare family days: a family empowerment programme

**DOI:** 10.1186/1750-1172-7-S2-A34

**Published:** 2012-11-22

**Authors:** Ane Lind, Lene Jensen, Birthe B Holm

**Affiliations:** 1 Rare Disorders Denmark, Copenhagen, DK-1220, Denmark

## 

Having a seriously ill child is intrusive for the entire family. Still, knowledge about how to manage family life for these families is limited, especially when the diagnosis is rare. To offer tools to cope with their situation, Rare Disorders Denmark (RDD) developed Rare Family Days (RFD).

RFD is an empowerment programme targeted families with children carrying a rare disease. The programme purpose is to empower families in their everyday life by offering an opportunity to create supportive networks, a forum for exchange of experience-based knowledge, knowledge of social and legal affairs, knowledge of psychological aspects of family life and tools for conflict management and goal setting.

RFD consists of:

- a weekend course for the entire family

- a one-day follow-up for parents

- access for parents to a closed internet forum with information, links and an opportunity to keep in contact

The programme form alternates between network-creating activities for the entire family, activities for children, activities for parents with professional presentations, presentations and discussions by volunteering patient representatives who engage throughout the programme as mentors for the families, group discussions and problem solving.

## Evaluation and effects

To document the effects, RFD was tested and evaluated based on a randomized controlled trial (RCT) supplemented with interviews with selected parents. Two groups of eight families completed the programme in 2011. The RCT was based on questionnaires for parents at baseline, after the weekend and after six month for the intervention group and a wait-list control.

As a proxy for empowerment, the parents’ knowledge of the child’s disease, the social welfare system and legal aspects were tested along benefits from networking and experience-based knowledge sharing.

Final results have not yet been reported. A full-length article is under preparation with expected submission deadline in December 2012. Preliminary results show that participators increased their knowledge in different areas, benefitted from creating network with each other and benefitted from sharing experience-based knowledge. The qualitative evaluation showed that families during experienced cohesion and unity. The physical meeting enabled them to learn from each other and from the instructors’ professional knowledge. Participation in RFD created drive and hope for the future.

